# Anterior Cervical Discectomy and Fusion Versus Cervical Disc Arthroplasty in Patients With Myelopathy and Radiculopathy: A Systematic Review and Meta-Analysis

**DOI:** 10.7759/cureus.94847

**Published:** 2025-10-18

**Authors:** Mohamed Elgamal, Ahmed Elnewishy, Muawia Yousif Fadlelmola Mohamed, Hatem Hussein, Mustafa Mouhsen, Dhanunjaiah Sure, Persidiu Iancu, Abdel Reda, Abuamar Zaidan, Zaher Dannawi

**Affiliations:** 1 Trauma and Orthopaedics, Southend Hospital, Southend, GBR; 2 Trauma and Orthopaedics, Royal Berkshire Hospital, Reading, GBR; 3 Orthopaedics and Trauma, Southend University Hospital NHS Foundation Trust, Southend, GBR; 4 Trauma and Orthopaedics, Southend University Hospital, Southend, GBR; 5 Trauma and Orthopaedics, Mid and South Essex NHS Foundation Trust, Southend, GBR; 6 Orthopaedic Surgery, Southend Hospital, Mid and South Essex NHS Foundation Trust, Southend, GBR; 7 Orthopaedics, Mid and South Essex NHS Foundation Trust, Southend, GBR; 8 Orthopaedics and Trauma, Mid and South Essex NHS Foundation Trust, Southend, GBR; 9 Spine Surgery, Southend Hospital, Mid and South Essex NHS Foundation Trust, Southend, GBR

**Keywords:** adjacent segment disease, anterior cervical discectomy and fusion, cervical disc arthroplasty, neck disability, randomized controlled trials, reoperation

## Abstract

Cervical disc arthroplasty (CDA), which preserves motion, has emerged as a viable alternative to anterior cervical discectomy and fusion (ACDF) for treating individuals suffering from cervical degenerative disc disease. The present meta-analysis was undertaken to assess both clinical and radiographic results comparing CDA against ACDF. A systematic review of randomized controlled trials (RCTs) was conducted following PRISMA (Preferred Reporting Items for Systematic Reviews and Meta-Analyses) guidelines. The analysis examined Neck Disability Index (NDI), Visual Analog Scale (VAS) measurements for cervical and upper extremity pain, frequencies of revision surgery, and adjacent segment disease (ASD). Random-effects models calculated pooled effect estimates. The I² statistic quantified heterogeneity, while funnel plot examination and Egger's test evaluated publication bias. The analysis incorporated 11 RCTs encompassing 2,537 patients. CDA demonstrated significant NDI improvement (standard mean difference (SMD) = -0.54, 95% CI: -1.00 to -0.08, p = 0.02, I² = 89%), neck pain reduction (SMD = -0.58, p = 0.004, I² = 86%), and arm pain alleviation (SMD = -0.44, p = 0.006, I² = 76%) relative to ACDF. Reoperation frequencies decreased with CDA (OR = 0.40, p = 0.0004, I² = 70%), alongside substantially diminished ASD risk (OR = 0.36, p < 0.00001, I² = 67%). Publication bias remained undetected. In conclusion, compared to ACDF, CDA provides superior outcomes in pain relief, functional recovery, and prevention of reoperation and ASD. CDA should be considered a preferred option in suitable patients.

## Introduction and background

Cervical degenerative disc disease (CDDD) represents a frequently encountered spinal condition, particularly among individuals in middle and advanced age groups. This problem is a degenerative progression associated with aging, affecting almost 95% of the adult population over 65 years old, and commonly presenting with neck pain, radiculopathy, or myelopathy, depending on the degree and location of the disc degeneration [1]. The ubiquitous nature of this problem leads to substantial health care costs related to both conservative and surgical interventions [2].

Recent epidemiological studies suggest that the burden of CDDD healthcare vastly affects the working population, especially employees and highly educated individuals, which seems related to long periods of sitting and/or work-related injury. A study in an Iranian population showed that about 49% of surgical treatments were at the C5-C6 level, confirming international literature reporting that biomechanical stress concentration occurs at C5-C6 [2,3].

In addition to the physical manifestations, CDDD's consequences extend to the dimensions of the quality of life and healthcare resource use for potentially affected persons, including extended treatment durations. Patient pathways to spinal specialists typically end in surgery, and large percentages of patients will ultimately need an anterior cervical discectomy and fusion (ACDF) or cervical disc arthroplasty (CDA) to correct their ongoing pain and/or neurologic deficits [4]. These patterns highlight the significance of early identification and patient-specific approaches to limit functional deficits and healthcare resource use [5].

Nerve root compression or irritation within the cervical spine is the cause of cervical radiculopathy, which most often leads to intervertebral disc herniation, degenerative spondylotic changes, and narrowing of the neural foramen. The nerve root involved undergoes inflammation and vascular compromise, leading to dermatomal patterns of radicular pain, sensory alterations, and motor weakness. Compressive pathology commonly occurs along with biochemical irritation by inflammatory mediators that sensitize nerve roots and enhance clinical presentation [6]. For many, conservative care will provide adequate resolution of symptoms, but for those with progressive or static neurologic function, surgical decompression is necessary [7].

Spinal cord compression characterizes cervical myelopathy, typically presenting with extensive neurological impairment encompassing ambulatory dysfunction, manual dexterity loss, and pathological reflexes. Compression may be fixed or variable in nature, with contributing mechanisms including vascular compromise, ischemic injury, and mechanical cord distortion [6]. Progressive deterioration typically characterizes this pathology, with surgical intervention generally necessary for resolution [8].

The overlap of radiculopathic and myelopathic presentations, which is called myeloradiculopathy, happens often. The presence of a combined upper and lower motor neuron sign is a diagnostic and treatment generator of complexity. Studies report that in patients with cervical myelopathy, concurrent radiculopathy is present in more than 50% of patients, adding complexity to the clinical picture [9]. A deep understanding of the anatomical and pathophysiological bases underpinning each condition is necessary for making safe and timely surgical decisions [10].

ACDF is the preferred surgical method for managing CDDD when radiculopathy or myelopathy occurs despite conservative care. It involves removal of the diseased disc followed by arthrodesis of the vertebral bodies to achieve spinal stability and neural decompression [11]. The fusion is optimized with PEEK (polyetheretherketone) cages or bone graft materials with a plate and screw construct for internal stabilization [12].

The effectiveness of ACDF is well established for neck pain, arm pain, and neurologic symptoms. A study of 50 subjects found that 64% experienced significant improvement at a one-year follow-up, with the greatest effect among patients who reported less than six months of symptoms before intervention [13].

However, the main disadvantage of ACDF is the high rate of adjacent segment disease (ASD) due to altered spinal kinematics after fusion. There is evidence that rigid fixation may place increased mechanical stress on adjacent discs, leading to degenerative changes and symptoms [14].

CDA offers a motion-preserving approach distinct from ACDF in treating CDDD. Through the implantation of artificial discs at deteriorated spinal segments, CDA maintains the natural biomechanics of the cervical spine, contrasting with fusion procedures. Preserving motion may lessen biomechanical stress on adjacent segments and may prevent or delay the incidence of a recognized fusion complication [15].

Research demonstrates that CDA achieves comparable or enhanced results relative to ACDF regarding alleviation of pain, restoration of function, and neurological improvement. Prospective studies have demonstrated that CDA patients had more favorable results than ACDF patients based on the Neck Disability Index (NDI), Visual Analog Scale (VAS), and Japanese Orthopedic Association (JOA), particularly single-level disease [16].

One distinct benefit of CDA is its potential of lowering ASD risk compared to fusion surgeries, where adaptive spinal kinematics can initiate the development of this complication. CDA reduces the risk of aberrant forces on adjacent discs by maintaining segmental motion, which potentially averts or slows degenerative changes at adjacent levels [17]. CDA is only appropriate when appropriate patient selection is performed and is usually indicated for younger patients with single-level pathology absent significant spondylosis or instability.

## Review

Objective

This systematic evaluation seeks to compare clinical results from ACDF versus CDA in patients diagnosed with cervical myelopathy and/or radiculopathy.

Methods

Search Strategy

A comprehensive search conducted in September 2025 examined PubMed, Scopus, Google Scholar, and the Cochrane Library to identify investigations comparing CDA with ACDF in patients with cervical radiculopathy and/or myelopathy. The search methodology integrated MeSH terminology and pertinent keywords, including "cervical disc arthroplasty", "CDA", "total disc replacement", "artificial cervical disc", "anterior cervical discectomy and fusion", "ACDF", "cervical degenerative disc disease", "radiculopathy", and "myelopathy". Boolean operators (AND, OR) refined search specifications, with restrictions applied to English-language human studies. The reference lists of included publications and relevant systematic reviews were manually screened to identify additional qualifying research.

Inclusion Criteria

Inclusion criteria encompassed randomized controlled trials (RCTs) that directly evaluated CDA against ACDF. Study populations consisted of adult patients with CDDD manifesting as radiculopathy, myelopathy, or both conditions simultaneously. Investigations required documentation of at least one primary clinical endpoint, such as pain amelioration or functional enhancement, utilizing validated assessment tools. English-language full-text articles represented the exclusive acceptable publication format.

Exclusion Criteria

Publications lacking direct CDA versus ACDF comparisons were eliminated from consideration. Case reports, case series, conference proceedings, narrative reviews, commentaries, and editorials did not meet inclusion standards. Research without retrievable outcome information or published in non-English languages was excluded.

Outcome Measures

Primary endpoints comprised functional enhancement, evaluated using established instruments, including the NDI [18], and pain amelioration, determined using VAS measurements for cervical and upper extremity pain [19]. Secondary endpoints encompassed range of motion (ROM) maintenance at treated segments, repeat surgery at index or contiguous levels, ASD development [20], patient-reported satisfaction measures, and procedure-associated complications such as heterotopic ossification (HO), pseudarthrosis, and implant failure.

Data Extraction and Quality Assessment

Data extraction was conducted by two reviewers working independently through standardized collection forms. Information gathered included study methodology, participant numbers, demographic characteristics, intervention details, outcome metrics, observation periods, and documented complications. Reviewer disagreements underwent resolution via discussion, with third-party arbitration when consensus proved unattainable. Methodological quality evaluation utilized the Cochrane Risk of Bias Tool 2.0 (RoB 2) [21], examining key areas including randomization procedures, protocol adherence, data completeness, measurement validity, and reporting selectivity. Quality evaluations categorized investigations as demonstrating low risk, some concerns, or high risk of bias.

Statistical Analysis

Statistical analyses were conducted with Review Manager (RevMan) software, version 5.4 (The Cochrane Collaboration, London, UK) [22]. Analysis of continuous variables, including NDI, VAS ratings for cervical discomfort, and VAS measurements for upper extremity discomfort, employed standardized mean differences (SMDs) accompanied by 95% confidence intervals (CIs). Dichotomous variables, such as rates of subsequent surgery and the occurrence of ASD, were examined using odds ratios (ORs) with 95% CIs. To account for variability across investigations, random-effects models were consistently implemented. The I² statistic served to evaluate statistical heterogeneity, where measurements exceeding 50% signified considerable heterogeneity. Assessment of potential publication bias involved visual inspection of funnel plots combined with Egger's regression test, establishing statistical significance at p < 0.05.

Results

Search and Study Selection

Multiple database explorations using systematic methodology identified research directly evaluating CDA against ACDF for individuals presenting with cervical radiculopathy and/or myelopathy. Database retrieval yielded 642 citations, of which 102 duplicate entries were removed before screening commenced. The title and abstract review encompassed 540 records. Among these, 478 investigations failed to satisfy established eligibility requirements demanding RCTs that assessed clinical outcomes comparing CDA with ACDF among adult populations diagnosed with CDDD.

**Figure 1:**
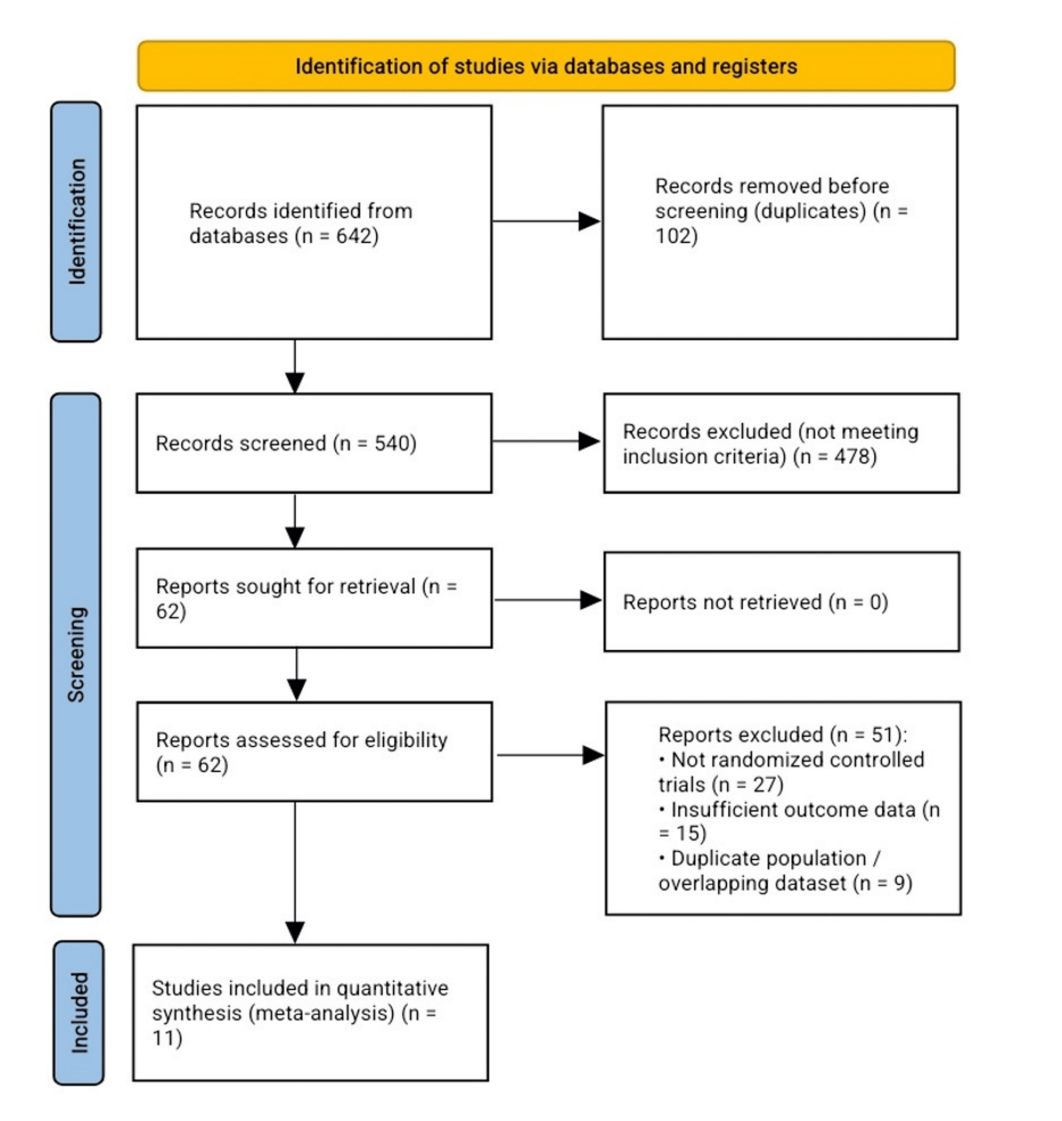
PRISMA flow chart for the included studies PRISMA: Preferred Reporting Items for Systematic Reviews and Meta-Analyses

A total of 62 publications underwent a comprehensive full-text examination. Subsequent detailed review resulted in 51 exclusions based on specific deficiencies: 27 lacked RCT design, 15 contained inadequate outcome information, and nine presented redundant populations or replicated data. The quantitative synthesis (meta-analysis) included 11 studies that met all established inclusion criteria. The complete study selection process is illustrated in Figure 1.

Study Characteristics

A total of 11 RCTs that directly evaluated CDA against ACDF among individuals suffering from cervical radiculopathy and/or myelopathy were incorporated into this systematic review and meta-analysis. The total pooled sample size across studies was 2,537 patients (CDA = 1,494; ACDF = 1,043), with individual study populations ranging from 44 to 416 participants. All included studies were rated as Level I evidence, consistent with the Oxford Centre for Evidence-Based Medicine classification. The methodological quality was generally high, with minimal risk of bias reported across domains such as randomization, outcome measurement, and reporting completeness.

The study population demonstrated moderate demographic variation. Average participant age ranged from 44.2 to 47.6 years across the included trials, while gender composition showed a relatively equal distribution, with male participants comprising 42% to 56% of the study samples. The majority of individuals exhibited CDDD affecting either one or two spinal levels, with the most commonly involved segments being C5-C6 and C6-C7. CDA interventions employed various FDA-approved implants, including Mobi-C, Prestige LP, PCM, Bryan, Discover, Kineflex|C, and ProDisc-C, with either single-level or two-level arthroplasty performed depending on the study. ACDF procedures typically utilize structural allografts or autografts with anterior plating systems.

Follow-up periods demonstrated considerable variation, spanning 24 months to 10 years, though the majority of investigations documented findings at five-year intervals or beyond. Clinical outcomes were assessed using standardized measurement tools across all studies, including the NDI, VAS for evaluating both cervical and upper extremity pain, and the Short Form-12 (SF-12) for examining physical and mental health components. Additional variables analyzed included mobility at the treated level (ROM), frequency of revision surgery, and development of ASD or HO.

The majority of investigations documented motion preservation at the treated level following CDA, accompanied by reduced adjacent-level degeneration and secondary surgery frequencies relative to ACDF. Although certain trials identified increased HO occurrence within CDA populations, functional outcomes remained largely unaffected across most investigations. The comprehensive analysis of included studies provides a robust foundation for quantitatively assessing the relative efficacy and safety characteristics of CDA versus ACDF (Table 1).

**Table 1:** Study design, sample size, level of evidence, patient demographics, interventions, follow-up duration, outcomes, and complications CDA: Cervical Disc Arthroplasty; ACDF: Anterior Cervical Discectomy and Fusion; DDD: Degenerative Disc Disease; RCT: Randomized Controlled Trial; FDA IDE: Food and Drug Administration Investigational Device Exemption; NDI: Neck Disability Index; VAS: Visual Analog Scale; SF-12: 12-Item Short Form Survey; PCS: Physical Component Summary; MCS: Mental Component Summary; ROM: Range of Motion; ASD: Adjacent Segment Disease; HO: Heterotopic Ossification; AE: Adverse Events; NS: Not Significant; TDR: Total Disc Replacement; FU: Follow-up; MPQ-DLV: Dutch Language Version of the McGill Pain Questionnaire; DSQ: Dysphagia Short Questionnaire; EQ-5D: EuroQol 5-Dimension Index; HADS: Hospital Anxiety and Depression Scale; RASP: Radiographic Adjacent Segment Pathology; CASP: Clinical Adjacent Segment Pathology; PPS: Posterior Probability of Superiority

Study	Study Design	Sample Size	Level of Evidence	Patient Demographics	Intervention Details	Follow-up Duration	Outcome Measures	Results	Complications	Conclusions
Coric et al. [23]	Prospective, randomized, multicenter FDA IDE trial (21 sites, single-level C3–C7 DDD with radiculopathy)	269 randomized: 136 CDA (Kineflex|C), 133 ACDF	I	Baseline balanced between groups (age, sex, BMI, NDI ≥40, VAS pain high, single-level pathology).	CDA: Kineflex|C metal-on-metal disc (SpinalMotion). ACDF: Structural allograft + anterior plate.	5 years (assessments at 6 weeks, 3, 6, 12, 24, 36, 48, 60 months)	Composite overall success (4- and 5-item FDA definitions), NDI, VAS neck/arm pain, ROM, HO, ASD, reoperation rates, AE profile, and serum ion levels (subset).	Overall Success (4-item): CDA 77.2% vs. ACDF 57.9% (p<0.05). Alternate 5-item composite: CDA was significantly greater at all time points except 36 months (p<0.05). NDI: Both groups improved by 6 weeks and maintained at 60 months (p<0.01). The between-group difference was not statistically significant (exact means not tabulated). VAS Neck/Arm Pain: Significant improvements in both groups, sustained through 60 months (p<0.01), with no significant group difference (exact means not tabulated). ROM: CDA showed increased ROM from baseline and maintained motion >6° at 60 months (p<0.01 vs. baseline). ACDF fused by definition (fusion rate 89.5%). Reoperations (index level): CDA 8.8% vs. ACDF 8.3% (NS). Adjacent Segment Degeneration: Superior level significantly less in CDA (p<0.01); inferior level NS. HO: Bridging ossification in 2.9% at 60 mo. Serum Ions (subset): Cobalt 0.21 µg/L, Chromium 0.31 µg/L—both well below thresholds of concern.	CDA: Device migration 1.4%, subsidence 1.4%, radiolucency 14.3%. Bridging HO 2.9%. ACDF: Fusion rate 89.5% at 5 years; radiolucency 7.0%; pseudarthrosis included in failure definition. AE and reoperation rates are similar (~8%).	Kineflex|C CDA was safe and effective at 5 years, with higher composite success and less superior-level ASD than ACDF. Both groups achieved durable improvements in pain and disability. CDA preserved motion, had low HO bridging, and had very low serum metal ion levels.
Davis et al. [24]	Prospective, randomized, controlled multicenter FDA IDE trial	330 randomized: 225 CDA (Mobi-C), 105 ACDF	I	Mean age: CDA 45.3 ± 8.1 years; ACDF 46.2 ± 8.0 years; Sex: CDA 50.2% male, 49.8% female; ACDF 42.9% male, 57.1% female; BMI: CDA 27.6 ± 4.5, ACDF 28.1 ± 4.2. Groups balanced	CDA: Mobi-C (mobile-bearing cobalt-chromium-molybdenum with UHMWPE insert); ACDF: corticocancellous allograft + anterior cervical plate	48 months (4 years)	NDI, VAS neck/arm pain, SF-12 PCS/MCS, ROM, neurological status, satisfaction, return to work, reoperation rates, radiographic success, HO, adjacent-segment degeneration	NDI: Improvement CDA 36.5 ± 21.3 vs. ACDF 28.5 ± 18.3 (p=0.0048); Success rates: CDA 79.3%, ACDF 53.4% (p<0.0001). VAS Neck pain: CDA 53 ± 30 vs. ACDF 48 ± 29 improvement (NS but trend favorable). VAS Arm pain: CDA 56 ± 31 vs. ACDF 53 ± 31 (NS). SF-12 PCS: CDA +13 ± 12 vs. ACDF +10 ± 12 (p<0.05). SF-12 MCS: CDA +11 ± 12 vs. ACDF +10 ± 12 (NS). ROM: CDA maintained baseline ROM; ACDF showed significant ↓. Overall success: CDA 66.0% vs. ACDF 36.0% (p<0.0001). Return to work: CDA 46 ± 101 days vs. ACDF 67 ± 113 days (NS). Satisfaction: CDA 96.4% vs. ACDF 89.0% (p=0.033). Reoperation: CDA 4.0% vs. ACDF 15.2% (p<0.0001). Adjacent-segment degeneration: CDA 41.5% vs. ACDF 85.9% (p<0.0001).	CDA: Clinically relevant HO (Grade III–IV) in 25.6% of patients and 16.6% of treated levels. One migration reported <24 months; no device failures thereafter. ACDF: Pseudarthrosis in 8.6%; fusion not achieved in 14.8% at 48 months. Major complications: CDA 4.0% vs. ACDF 7.6% (NS).	Two-level CDA with Mobi-C was safe, maintained ROM, reduced disability and pain, had fewer reoperations, lower adjacent-segment degeneration, and superior overall success compared with ACDF at 4 years.
Donk et al. [25]	Prospective, double-blind, single-center RCT (PROCON trial), 3-arm design	142 randomized: 50 ACDA (Bryan disc), 47 ACDF (standalone cage), 45 ACD (no implant)	I	Mean age 44.9 ± 6.5 years; 50% female; baseline NDI ~18; distribution: C5–6 (66 cases), C6–7 (73 cases); groups balanced	ACD: standard discectomy only. ACDF: cage stand-alone (Brantigan cervical I/F, autologous bone). ACDA: Bryan disc prosthesis (Medtronic). No collars post-op; ACDA patients given meloxicam ×2 weeks	Median 8.9 ± 1.9 years (range 5.6–12.2)	NDI (primary), MPQ-DLV, VAS/NRS pain, SF-36 PCS/MCS, reoperations, complications	NDI: Baseline ~18.8 → 7.5 ± 8.5 at 9 years (all groups improved; p=0.009 vs. baseline). No significant difference among ACD, ACDF, and ACDA (p=0.324). Good outcome (NDI ≤7 at final FU): ACDA 73.5%, ACDF 60.9%, ACD 57.8% (NS, p=0.239). SF-36: PCS improved +32.1 ± 2.5; MCS improved +22.8 ± 2.1; no difference between groups (p>0.8). NRS pain (final FU, n=140): Arm 1.8 ± 2.5; Neck 1.9 ± 2.6; no group difference (p=0.622/0.496). VAS (MPQ-DLV): Improved (moment 17.3 ± 24.0); no group difference (p>0.4). Reoperations (index/adjacent): 11 patients (7.8%) – 3 index, 8 adjacent; NS among groups (p=0.132).	ACDA dysphagia 4%; ACDF dysphagia 9%.	A long-term (≈9 years) RCT found no significant differences in NDI, pain, or QoL among ACD, ACDF, and ACDA. All groups improved rapidly within 6 weeks and remained stable. ACDA showed a trend toward more “good outcomes” (NDI ≤7) and fewer adjacent-level surgeries, but differences were not statistically significant. Study underpowered; conclusions considered inconclusive.
Gornet et al. [26]	Prospective, randomized, multicenter, FDA IDE + post-approval trial	209 CDA (Prestige LP) vs. 188 ACDF	I	Mean age: CDA 47.1 ± 8.3 years, ACDF 47.6 ± 8.4 years; Sex: CDA 56% female, ACDF 54% female; Presenting: Radiculopathy ~72%, Radiculopathy + Myelopathy ~24% in both groups	CDA: Prestige LP cervical disc (titanium ceramic composite, Medtronic), 2-level implantation (C5–C7). ACDF: Cortical ring allograft + ATLANTIS anterior cervical plate (Medtronic).	10 years	Overall success (FDA composite: NDI improvement ≥15 pts, no reop, no major AE, stable neuro exam), NDI, VAS neck/arm pain, SF-36 PCS/MCS, ROM, radiographic fusion, patient satisfaction	Overall Success: CDA 80.4% vs. ACDF 62.2% (PPS=99.9%). NDI Success: CDA 88.4% vs. ACDF 76.5% (PPS=99.5%). Neuro Success: CDA 92.6% vs. ACDF 86.1% (PPS=95.6%). Pain/Disability: CDA had superior improvements in NDI (PPS=99.9%) and neck pain (PPS≈100%). Arm pain & SF-36 PCS better numerically in CDA, not always statistically significant. ROM: Maintained at treated levels in CDA; ACDF fused (fusion rate >93%). Reoperations: Index-level: CDA 4.7% vs. ACDF 17.6% (p<0.001). Adjacent-level: CDA 9.0% vs. ACDF 17.9% (p<0.05).	CDA: Severe HO (Grade III–IV) in 39% at 10 years (Grade IV sup 8.2%, inf 10.3%); no device failures; fewer serious device-related AEs (3.8% vs. 8.1%). ACDF: Higher rates of pseudoarthrosis, nonunion (up to 5%), and higher reoperation rates	At 10 years, 2-level CDA with Prestige LP maintained motion, provided superior NDI, neck pain, neurological outcomes, and had fewer serious Aes and secondary surgeries compared to ACDF. CDA was a safe and effective long-term alternative to fusion.
Hisey et al. [27]	Prospective, randomized, controlled, multicenter FDA IDE trial (23 centers, 2:1 randomization)	245 randomized: 164 TDR (Mobi-C) vs. 81 ACDF	I	Single-level symptomatic cervical DDD (C3–C7) with radiculopathy/myeloradiculopathy; baseline demographics balanced	CDA: Mobi-C® cervical disc prosthesis (LDR Medical, unconstrained mobile-bearing). ACDF: corticocancellous allograft + anterior plate (Slim-Loc, Sofamor Danek Atlantis).	5 years (60 months)	Primary: Composite success (NDI ≥30/100 improvement, no reop, no device AE, no neuro decline, no treatment change). Secondary: NDI, VAS neck/arm pain, SF-12 PCS/MCS, ROM, patient satisfaction, ASD, HO, reoperations	Overall success: TDR 61.9% vs. ACDF 52.2% (non-inferior). NDI success: TDR 68.1% vs. ACDF 62.1%. VAS Neck pain: both improved from ~70 preop → <20 at 5 years, no significant difference. VAS Arm pain: both improved significantly, stable over 5 years. SF-12 PCS: Preop 32.5 → 47.6 TDR, 33.8 → 48.3 ACDF (similar). Satisfaction: 92.0% TDR vs. 83.9% ACDF, very satisfied. Recommend surgery: 97.1% TDR vs. 91.1% ACDF would recommend. Subsequent surgery: TDR 4.9% vs. ACDF 17.3% (p<0.01). Device-related reops: TDR 3.0% vs. ACDF 11.1% (p<0.02). ROM: maintained in TDR (~7°), fused in ACDF (<2°). ASD: Superior ASD: TDR 37.1% vs. ACDF 54.7% (p<0.03). Inferior ASD: significantly lower in TDR at all time points. HO: Grade 4 in 8.5% at 5 years	CDA: HO leading to fusion in 8.5%; some device removals/revisions (oversized, HO-related pain, malpositioned). ACDF: Pseudarthrosis reoperations, higher ASD reops, hardware issues. Both groups had low device migration (none reported).	TDR with Mobi-C was non-inferior to ACDF at 5 years, with advantages of preserved motion, lower reoperation rates, and less adjacent segment degeneration. Both procedures are safe and effective, but CDA showed potential long-term benefits.
Loumeau et al. [28]	Prospective randomized single-site IDE trial	44 randomized (22 CDA, 22 ACDF) + 19 continued access CDA	I	Single-level C4–C7; groups balanced age/sex	CDA: ProDisc-C; ACDF: allograft + plating	7 years (84 months)	NDI, VAS neck/arm, SF-36, ROM, satisfaction	NDI: CDA 53.95 ± 11.93 → 15.44 ± 19.59; ACDF 53.64 ± 14.11 → 25.47 ± 23.96 (p=0.015) VAS Neck: CDA 80.0 ± 10.8 → 11.7 ± 18.8; ACDF 73.9 ± 16.6 → 28.0 ± 34.7 (p=0.007) VAS Arm: CDA 74.6 ± 24.1 → 12.6 ± 23.4; ACDF 71.7 ± 25.4 → 27.3 ± 35.7 (p=0.027) ROM: CDA maintained >7° flex–ext, 4.4° lateral; ACDF ↓ significantly (p<0.001) Satisfaction: >80% in both, higher in CDA Reoperations: ACDF 6/22 (27.3%), CDA 0	CDA: HO (Grade III 44%, Grade IV 15%); no device failures. ACDF: pseudoarthrosis, 4 adjacent level surgeries	CDA maintained motion, reduced pain/disability, and eliminated reoperations at 7 years compared with ACDF
MacDowall et al. [29]	Prospective, multicenter, randomized controlled superiority trial (Sweden, 3 centers)	153 (83 CDA, 70 ACDF)	I	Mean age: ADR 46.9 ± 6.8 years, Fusion 47.0 ± 6.9 years; Sex: ADR 42M/41F, Fusion 33M/37F; Smokers: ADR 32%, Fusion 31%; Symptom duration: >2 years in ~40% of both groups; Levels: C5–6 (33%), C6–7 (33%), C5–7 (29%)	ADR: Discover cervical disc (DePuy Synthes, unconstrained ball-and-socket, titanium endplates + UHMWPE core); NSAIDs x10 days post-op to reduce HO. Fusion: Iliac crest autograft + anterior titanium plate. Standard Smith-Robinson approach; patients/surgeons blinded until decompression is completed	5 years (66 mo, range 57–77)	Primary: NDI. Secondary: EQ-5D, VAS neck/arm pain, DSQ (dysphagia), HADS (anxiety/depression). Tertiary: neurological exam, ROM (Cobb angle), HO, MRI-assessed ASP (RASP/CASP), secondary surgery	NDI: ADR 64 → 36; Fusion 61 → 32 (Δ=2.5; 95% CI −4.5 to 9.4; p=0.48). Both ~50% reductions. EQ-5D: ADR 0.37 → 0.62; Fusion 0.46 → 0.72 (NS). VAS Neck: ADR 57 → 29; Fusion 59 → 32 (NS). VAS Arm: ADR 57 → 24; Fusion 57 → 24 (NS). DSQ (dysphagia): ADR 1.4 vs. Fusion 2.3 at 5 years (p=0.045). HO: 25% spontaneous fusion; 41% Grade III; men > women (p=0.001). Motion preserved: 54% ADR ≥5° ROM at index level. ASP: CASP severe requiring surgery: 5 ADR vs. 5 Fusion (NS). RASP progression: similar between groups. Reoperations: ADR 21% vs. Fusion 10% (p=0.11). HR for women ADR vs. Fusion = 10.5 (95% CI 1.4–80.9); HR for men 0.55 (NS)	ADR: Higher secondary surgery (loosening, subsidence, spontaneous fusion). HO common (25% fused, 41% Grade III). Facet arthropathy progression noted. Sex differences: women have a higher risk of reoperation. Fusion: Lower reoperation risk, but donor-site morbidity from iliac crest graft.	No clinical or radiological advantage of ADR over fusion at 5 years. Both groups halved NDI and improved equally in PROs. ADR preserved motion in ~50% but did not prevent ASP. Women in the ADR group had a significantly higher risk of reoperation. Fusion with autograft had stable results and fewer revisions.
Nunley et al. [30]	Prospective, randomized clinical trial (3 high-enrolling sites from the original FDA IDE Mobi-C trial)	155 enrolled (105 CDA, 50 ACDF). At 10 years: 107 followed (69%).	I	Mean age: CDA 44.2 ± 8.0 years, ACDF 43.9 ± 8.2 years; ~50% female; BMI ~27.5; 46.7% 1-level, 53.3% 2-level; levels: C5–6 (most common), C6–7. Baseline NDI ~50, VAS neck pain ~72–73, VAS arm pain ~45–48. Groups balanced	CDA: Mobi-C cervical disc (Zimmer Biomet, cobalt-chrome endplates + UHMWPE mobile core). ACDF: Structural allograft + anterior plate fixation.	10 years (range up to 13.1 years). Original surgeries 2006–2008.	Composite overall success (≥15-point NDI improvement, no reoperation, no serious AE, maintained neuro status), NDI, VAS neck/arm pain, SF-12 PCS/MCS, patient satisfaction, neurologic success, adjacent-segment pathology (radiographic & clinical), reoperation rates	Composite success (10 years): CDA 62.4% vs. ACDF 22.2% (p<0.0001). Subsequent surgery (any): CDA 7.2% vs. ACDF 25.5% (p=0.001). Adjacent-level surgery: CDA 3.1% vs. ACDF 20.5% (p=0.0005). Radiographic Adjacent-Segment Pathology (Grade 3/4 RASP): CDA 12.9% vs. ACDF 39.3% (p=0.006). NDI success: CDA 84.8% vs. ACDF 74.1% (NS, p=0.25). Mean NDI change: CDA –34.1 vs. ACDF –30.0. VAS Neck pain: CDA –59.4 vs. ACDF –53.4 (NS, p=0.25). VAS Arm pain: CDA –56.5 vs. ACDF –47.4 (NS, p=0.13). SF-12 PCS: CDA +15.7 vs. ACDF +9.5 (p=0.004). SF-12 MCS: CDA +9.1 vs. ACDF +7.2 (NS, p=0.44). Neurologic success: CDA 87.8% vs. ACDF 55.6% (p=0.0015). Satisfaction: CDA 89.9% vs. ACDF 85.2% (p=0.50); “very satisfied”: CDA 98.7% vs. ACDF 88.9% (p=0.05)	CDA: Treatment-related AEs 18.6% vs. ACDF 32.2% (p=0.024). Events: subsidence (2 cases), radiculopathy (1), posterior fusion required in 1 case. ACDF: AEs included kyphosis, decreased ROM, diminished reflexes, muscle spasms (none required reoperation). Index-level reoperation: CDA 5.2% vs. ACDF 10.5% (NS, p=0.18)	At 10 years, Mobi-C CDA showed superior outcomes vs. ACDF: higher composite success, fewer reoperations, less adjacent-level surgery, and better neurologic success. Both groups improved in NDI, VAS, and SF-12, but CDA maintained motion and reduced adjacent pathology. CDA is a safe and effective long-term alternative to ACDF
Phillips et al. [31]	Prospective, multicenter, randomized FDA IDE trial	416 randomized (224 PCM, 192 ACDF); 218 PCM and 185 ACDF treated; 163 PCM vs 130 ACDF per protocol at 5 years; 68 PCM vs 42 ACDF at 7 years	I	Single-level C3–C7 DDD with radiculopathy ± myelopathy; Baseline characteristics balanced	CDA: PCM cervical disc (NuVasive, nonconstrained metal-polymer). ACDF: Allograft + anterior plate	5–7 years	NDI, VAS neck/arm, SF-36 PCS/MCS, neurological status, ROM, disc height, HO, dysphagia, satisfaction, reoperations	NDI: Mean at 5 years: PCM 20.4 vs. ACDF 28.5 (p=0.001). NDI success: PCM 85.0% vs. ACDF 74.2% (p=0.026). VAS Neck pain: PCM significantly better at 5 years (p=0.002). VAS Arm pain: No difference (p>0.05). SF-36 PCS: Improvement ≥15%: PCM 73.7% vs. ACDF 56.7% (p=0.004). SF-36 MCS: Similar (p=0.189). Neurological Success: PCM 92.4% vs. ACDF 87.5% (NS). Dysphagia: PCM 8.8 ± 15.7 vs. ACDF 16.9 ± 24.2 (p=0.001). Satisfaction: PCM 86.9 ± 21.6 vs. ACDF 78.3 ± 29.6 (p=0.005). ROM: PCM maintained 5.2° ± 3.8° vs. ACDF fused (0.5° ± 0.5°). Fusion: ACDF 94.4%. Adjacent-level degeneration: PCM 33.1% vs. ACDF 50.9% (p=0.006). Reoperations (SSSI): 5 years: PCM 8.1% vs. ACDF 12.0% (NS); 7 years: PCM 8.5% vs. ACDF 13.0% (NS, trend favoring PCM)	CDA: HO Grade III–IV in 12.7% at 5 years; 6 PCM removals (pain/migration, some trauma-related). ACDF: Pseudarthrosis 1 case; higher adjacent-level degeneration surgeries (11/14 late-term reoperations). Both groups had similar systemic AEs	PCM arthroplasty showed superior NDI, neck pain, PCS, satisfaction, lower dysphagia, and fewer adjacent-level degenerations compared with ACDF at 5–7 years. Both procedures are effective, but PCM preserved motion and reduced reoperation trends.
Radcliff et al. [32]	Prospective, randomized, controlled, multicenter FDA IDE trial (24 sites)	330 (225 CDA, 105 ACDF)	I	Mean age: CDA 45.3 ± 8.1 years, ACDF 46.2 ± 8.0 years; Sex: CDA 50.2% male, ACDF 42.9% male; Baseline NDI: CDA 53.9 ± 15.6, ACDF 55.4 ± 15.3; VAS Neck: CDA 71.2 ± 20.5, ACDF 74.6 ± 18.9	CDA: Mobi-C (cobalt-chromium endplates + UHMWPE core). ACDF: corticocancellous allograft + anterior plating.	60 months (5 years)	NDI, VAS neck/arm, SF-12 PCS/MCS, ROM, neurological exam, patient satisfaction, reoperation rates	NDI improvement: CDA −37 ± 20 vs. ACDF −28 ± 18 (p=0.0003). VAS Neck: CDA 18.7 ± 26.1 vs. ACDF 28.5 ± 28.8 at 60 months (NS). VAS Arm: CDA 11.9 ± 21.2 vs. ACDF 22.2 ± 27.4 at 60 months (NS). SF-12 PCS: CDA +13 vs. ACDF +9 (p=0.0073). Patient Satisfaction: CDA 96.4% vs. ACDF 89.5% (p=0.04). Recommend surgery again: CDA 94.8% vs. ACDF 84.2% (p=0.01). Overall success (FDA composite): CDA 61% vs. ACDF 31% (p<0.0001). Reoperations: CDA 7.1% vs. ACDF 21% (p=0.0006). Index-level: CDA 4.3% vs. ACDF 16.2%. Adjacent-level: CDA 3.1% vs. ACDF 11.4%. ROM: maintained in CDA (10.1° sup, 8.4° inf flex-ext); ACDF fused (90.5% fusion rate, 14% nonunion). ASD: CDA 50.7% vs. ACDF 90.5% (p<0.0001).	CDA: HO (Grade III–IV in 29.7%, Grade IV in 9.7%), 4 implant malpositions (1.7%). ACDF: 14% nonunion, 8.6% symptomatic pseudarthrosis needing surgery, and higher adjacent-level degeneration (70–90%). Dysphagia: similar (CDA 16%, ACDF 21%)	2-level CDA with Mobi-C showed superior NDI, PCS, satisfaction, and lower reoperation rates vs. ACDF at 5 years. CDA preserved motion and reduced adjacent-level degeneration. Both procedures were safe, but CDA provided better long-term outcomes in properly selected patients.
Rožanković et al. [33]	Prospective, single-center randomized trial	105 patients randomized (51 CDA with Discover, 50 ACDF; 4 lost to follow-up)	I	Mean age: CDA 41.3 ± 8.8 years (22–58), ACDF 41.9 ± 9.4 years (22–60); Sex: CDA 49% male, ACDF 50% male; Symptom duration: CDA 14.1 ± 12.7 mo, ACDF 14.4 ± 10.8 mo; Levels treated: mostly C5–C6 (≈53%), C6–C7 (≈36%)	CDA: Discover artificial cervical disc (DePuy Spine, ball-and-socket titanium alloy endplates + UHMWPE core). ACDF: Allograft fusion (DuoCage, SBM France). Both were performed via a standard anterior approach by a single surgeon.	24 months (minimum 2-year follow-up)	NDI, VAS neck and arm pain, ROM (dynamic X-rays), neurological status, radiographic fusion/prosthesis integrity	VAS Neck pain: Preop ~7.5 in both groups. At 24 mo: CDA 2.36 ± 0.75 vs. ACDF 3.46 ± 0.68 (p<0.001). VAS Arm pain: Preop ~7.7 in both groups. At 24 mo: CDA 1.70 ± 0.76 vs. ACDF 2.42 ± 0.57 (p<0.001). NDI: Preop CDA 50.9 ± 11.5 vs. ACDF 51.2 ± 8.6. At 24 mo: CDA 11.6 ± 4.4 vs. ACDF 19.7 ± 6.0 (p<0.001). ROM: Preserved in 92% of the CDA group; fusion rate 98% in the ACDF group.	CDA: 4 cases (8%) of heterotopic ossification → loss of ROM at treated level; 1 dural tear. ACDF: 1 case of graft collapse, migration & pseudoarthrosis requiring revision; 1 dural tear. No device migration in the CDA group	Discover CDA provided significantly better NDI and VAS outcomes at all follow-ups compared to ACDF, preserved ROM in most patients, and had fewer reoperations. ACDF had a higher risk of pseudoarthrosis. Both were safe, but CDA offered superior functional outcomes at 2 years.

Quality Assessment of the Included Studies

Evaluation of methodological rigor for the incorporated RCTs employed the Cochrane Risk of Bias Tool version 2.0 (RoB2; The Cochrane Collaboration, London, UK). Five critical domains underwent scrutiny through this assessment instrument: randomization procedures, deviations from intended interventions, missing outcome information, outcome assessment methods, and selection in reported findings. The studies were assigned comprehensive bias classifications of low risk, some concerns, or high risk following an aggregate assessment of these individual components.

**Figure 2:**
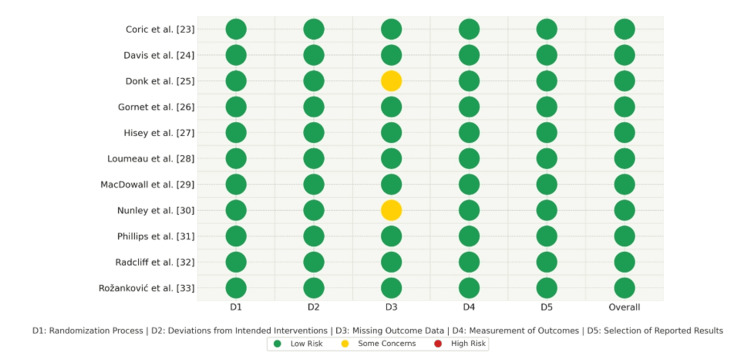
Systematic evaluation of potential bias among incorporated studies employing the Cochrane RoB2 tool

Most investigations exhibited low risk of bias for every assessed domain, reflecting robust methodological standards. Several trials raised some concerns, mainly due to participant loss during prolonged follow-up or incomplete outcome documentation. No studies received a high-risk designation. The predominance of low-risk ratings throughout the incorporated research strengthens confidence in the meta-analytic results. Table 2 provides comprehensive risk-of-bias evaluations, and Figure 2 displays these assessments visually.

**Table 2:** Quality assessment for included studies using the Cochrane Risk of Bias Tool 2.0 (RoB2)

Study	Randomization Process	Deviations From Intended Interventions	Missing Outcome Data	Measurement of Outcomes	Selection of Reported Results	Overall Risk of Bias
Coric et al. [23]	Low risk	Low risk	Low risk	Low risk	Low risk	Low risk
Davis et al. [24]	Low risk	Low risk	Low risk	Low risk	Low risk	Low risk
Donk et al. [25]	Low risk	Low risk	Some concerns (attrition, long FU)	Low risk	Low risk	Low risk
Gornet et al. [26]	Low risk	Low risk	Low risk	Low risk	Low risk	Low risk
Hisey et al. [27]	Low risk	Low risk	Low risk	Low risk	Low risk	Low risk
Loumeau et al. [28]	Low risk	Low risk	Low risk	Low risk	Low risk	Low risk
MacDowall et al. [29]	Low risk	Low risk	Low risk	Low risk	Low risk	Low risk
Nunley et al. [30]	Low risk	Low risk	Some concerns (long-term attrition)	Low risk	Low risk	Low risk
Phillips et al. [31]	Low risk	Low risk	Low risk	Low risk	Low risk	Low risk
Radcliff et al. [32]	Low risk	Low risk	Low risk	Low risk	Low risk	Low risk
Rožanković et al. [33]	Low risk	Low risk	Low risk	Low risk	Low risk	Low risk

Results of the meta-analysis

Comparison of CDA and ACDF for NDI Score

**Figure 3:**
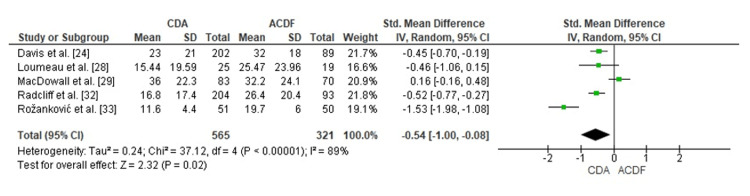
Forest plot comparing CDA and ACDF for NDI reduction score CDA: Cervical Disc Arthroplasty; ACDF: Anterior Cervical Discectomy and Fusion; NDI: Neck Disability Index; SMD: Standard Mean Difference; CI: Confidence Interval Source: [24,28,29,32,33]

Forest plot analysis examined CDA effectiveness relative to ACDF for neck disability reduction utilizing the NDI. Pooled SMD measured -0.54 (95% CI: -1.00 to -0.08), demonstrating statistically significant superiority for CDA (p = 0.02). Considerable heterogeneity emerged (I² = 89%), reflecting differences in study methodologies, patient characteristics, and prosthesis designs. Aggregated findings indicate greater CDA efficacy compared with ACDF for neck disability amelioration (Figure 3).

Publication Bias Assessment for NDI Score

**Figure 4:**
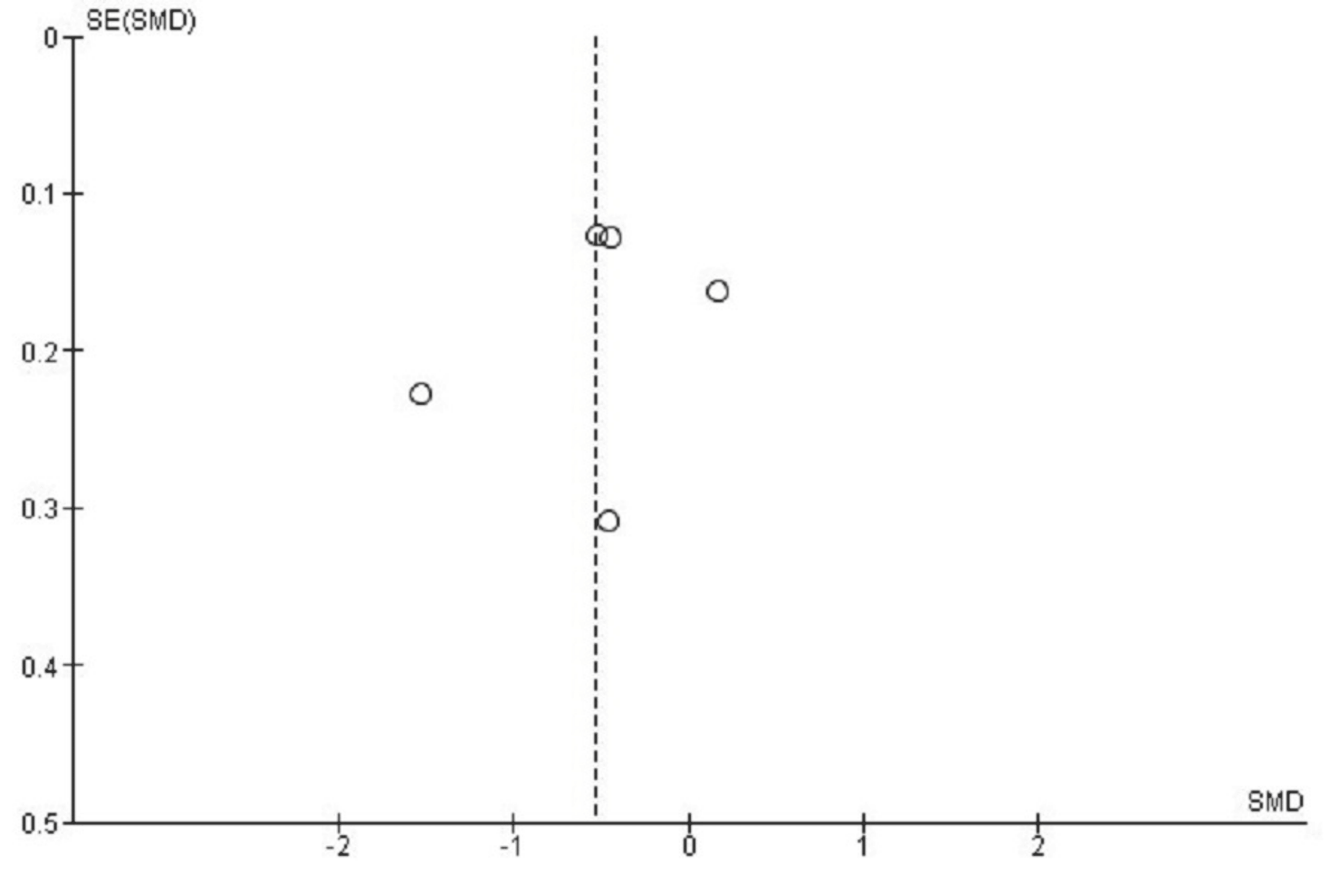
Funnel plot assessing publication bias for NDI outcomes in studies comparing CDA and ACDF CDA: Cervical Disc Arthroplasty; ACDF: Anterior Cervical Discectomy and Fusion; NDI: Neck Disability Index; SMD: Standard Mean Difference; SE: Standard Error

Funnel plot evaluation examining the NDI outcome for publication bias reveals evident distributional asymmetry, potentially reflecting inter-study heterogeneity or effects associated with smaller sample sizes. However, Egger's test produced non-significant findings (p > 0.05), suggesting that distributional asymmetry originated from inter-study heterogeneity rather than publication selectivity (Figure 4).

Comparison of CDA and ACDF for Neck Pain Reduction (VAS Score)

**Figure 5:**
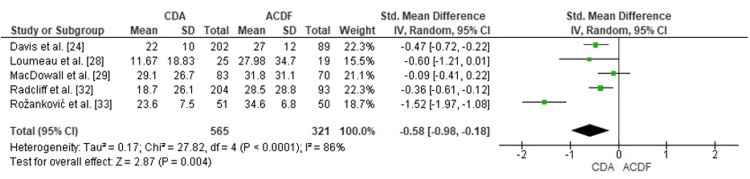
Forest plot comparing CDA and ACDF for neck pain reduction (VAS score) CDA: Cervical Disc Arthroplasty; ACDF: Anterior Cervical Discectomy and Fusion; VAS: Visual Analog Scale; SMD: Standard Mean Difference; CI: Confidence Interval Source: [24,28,29,32,33]

The forest plot assessment investigated CDA performance against ACDF regarding neck pain alleviation through VAS measurements. The combined SMD yielded -0.58 (95% CI: -0.98 to -0.18), revealing a statistically significant CDA advantage (p = 0.004). Notable heterogeneity appeared (I² = 86%), presumably attributable to variations in patient selection criteria, prosthesis specifications, and follow-up intervals. Cumulative evidence demonstrates superior neck pain reduction achieved through CDA versus ACDF (Figure 5).

Publication Bias Assessment for VAS Score

**Figure 6:**
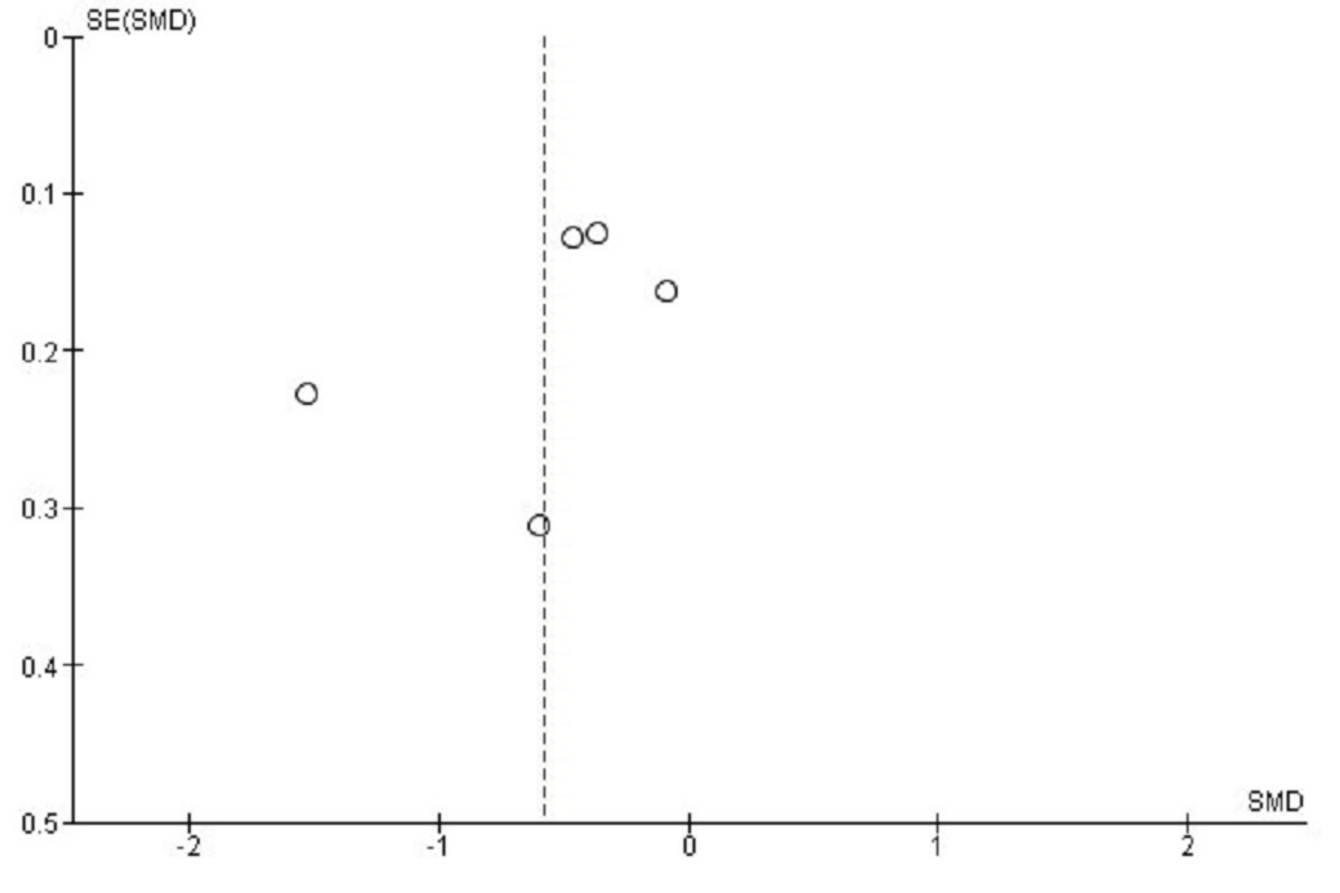
Funnel plot assessing publication bias for VAS outcomes in studies comparing CDA and ACDF CDA: Cervical Disc Arthroplasty; ACDF: Anterior Cervical Discectomy and Fusion; VAS: Visual Analog Scale; SMD: Standard Mean Difference; SE: Standard Error

The funnel plot evaluation for VAS neck pain outcomes demonstrated apparent asymmetry, possibly reflecting heterogeneity or small-study influences. Egger's test revealed no significant publication bias (p > 0.05), indicating that asymmetrical patterns resulted from differences in study methodology rather than selective publication (Figure 6).

Comparison of CDA and ACDF for Arm Pain Reduction (VAS Score)

**Figure 7:**
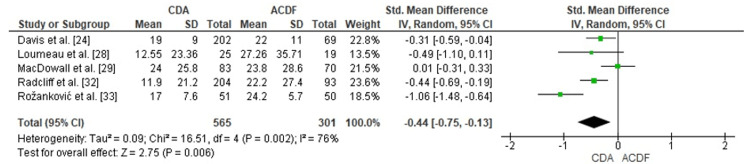
Forest plot comparing CDA and ACDF for arm pain reduction (VAS score) CDA: Cervical Disc Arthroplasty; ACDF: Anterior Cervical Discectomy and Fusion; VAS: Visual Analog Scale; SMD: Standard Mean Difference; CI: Confidence Interval Source: [24,28,29,32,33]

The forest plot examination assessed CDA efficacy versus ACDF for arm pain reduction measured via VAS. The aggregated SMD registered -0.44 (95% CI: -0.75 to -0.13), establishing statistically significant CDA benefit (p = 0.006). Moderate heterogeneity characterized the incorporated investigations (I² = 76%), suggesting variability in study populations or follow-up periods. The combined analysis confirms superior CDA performance relative to ACDF for arm pain alleviation (Figure 7).

Publication Bias Assessment for VAS Score

**Figure 8:**
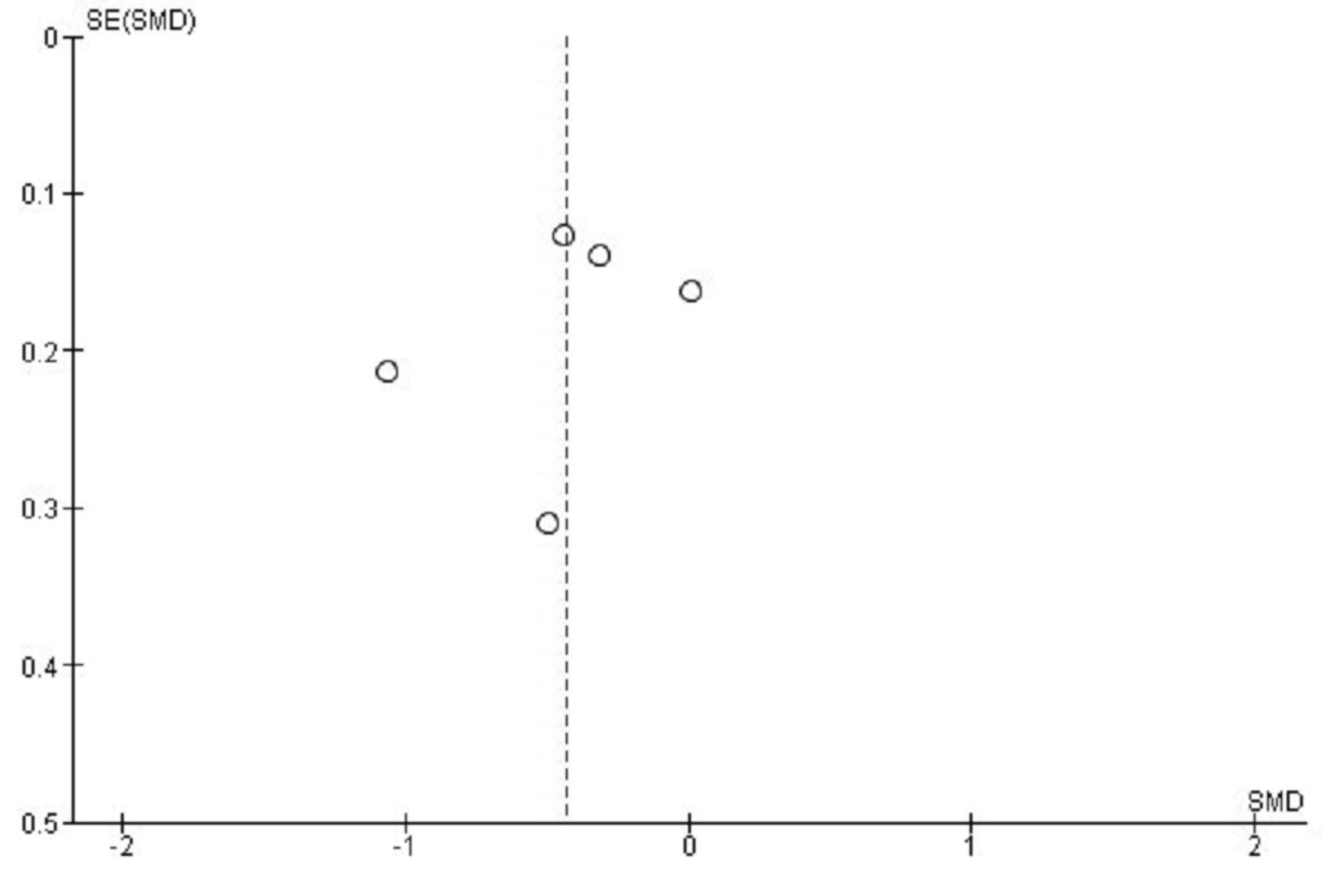
Funnel plot assessing publication bias for VAS outcomes in studies comparing CDA and ACDF CDA: Cervical Disc Arthroplasty; ACDF: Anterior Cervical Discectomy and Fusion; VAS: Visual Analog Scale; SMD: Standard Mean Difference; SE: Standard Error

There was some asymmetry in the funnel plot analysis of publication bias for VAS arm pain outcomes, which might have been caused by heterogeneity or small-study effects. Asymmetry most likely arises from inter-study variation rather than reporting bias, as shown by Egger's test, which found no significant publication bias (p > 0.05) (Figure 8).

Comparison of CDA and ACDF for Reoperation Rates

**Figure 9:**
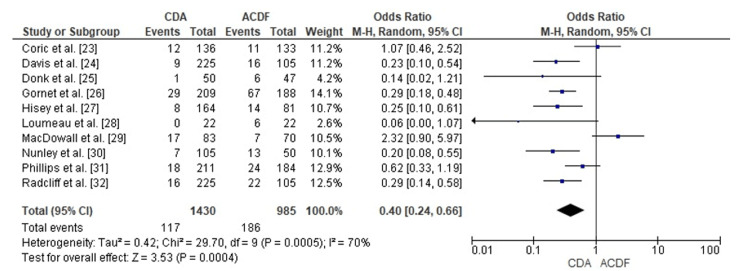
Forest plot comparing CDA and ACDF for reoperation rates CDA: Cervical Disc Arthroplasty; ACDF: Anterior Cervical Discectomy and Fusion; OR: Odds Ratio; CI: Confidence Interval Source: [23–32]

The forest plot analysis contrasted reoperation frequencies between CDA and ACDF. The combined OR measured 0.40 (95% CI: 0.24 to 0.66), demonstrating a statistically significant reoperation risk reduction following CDA (p = 0.0004). Moderate heterogeneity emerged among investigations (I² = 70%), potentially attributable to surgical technique variations, prosthesis specifications, or follow-up intervals. The aggregated findings demonstrate significantly reduced revision surgery frequencies for CDA compared to ACDF (Figure 9).

Publication Bias Assessment for Reoperation Rate

**Figure 10:**
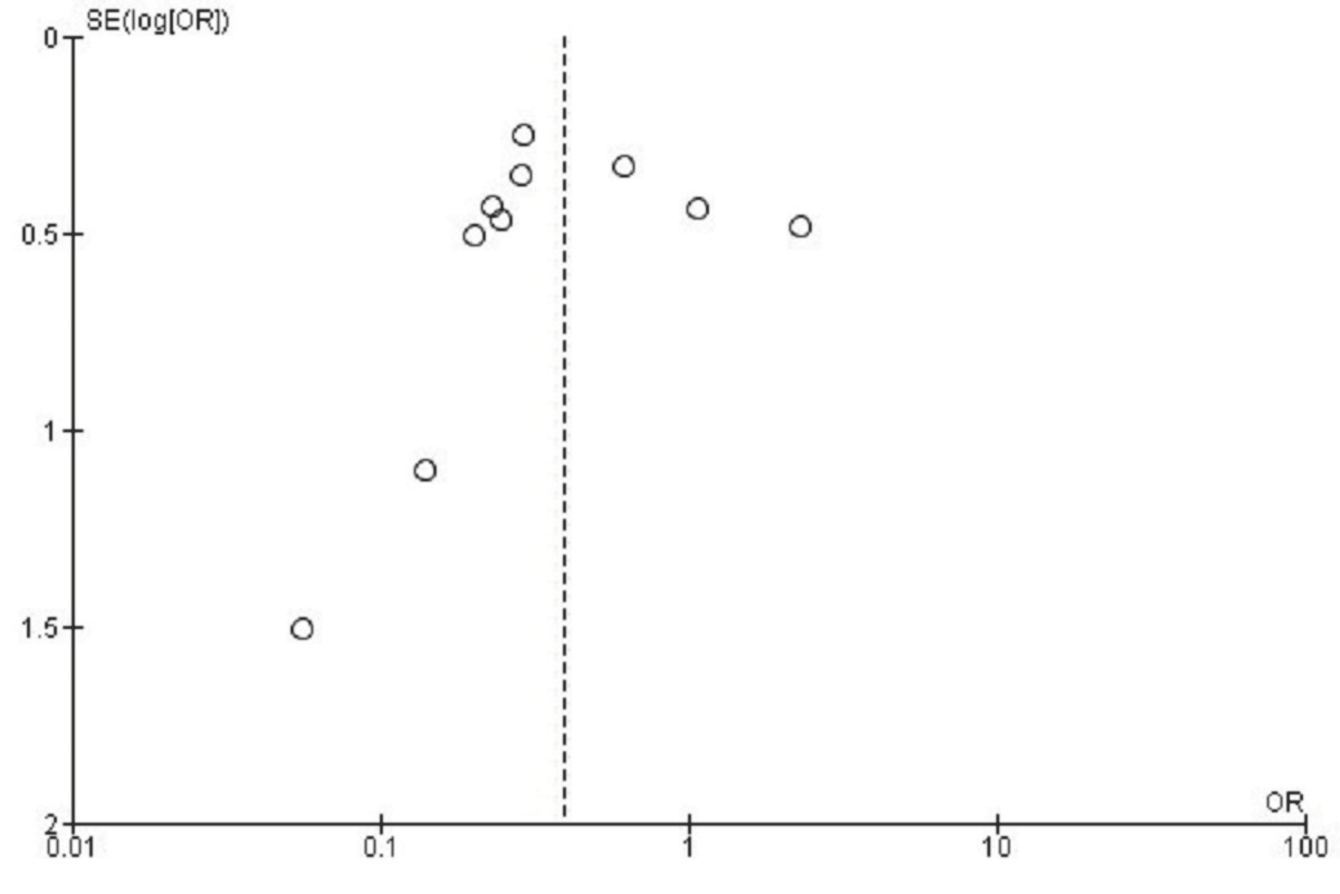
Funnel plot assessing publication bias for reoperation rates in studies comparing CDA and ACDF CDA: Cervical Disc Arthroplasty; ACDF: Anterior Cervical Discectomy and Fusion; OR: Odds Ratio; SE: Standard Error

The funnel plot examination for reoperation outcomes showed evident asymmetry, potentially reflecting heterogeneity or small-study influences. Egger's test revealed no significant publication bias (p > 0.05), indicating that asymmetrical patterns resulted from between-study differences rather than selective publication practices (Figure 10).

Comparison of CDA and ACDF for ASD

**Figure 11:**
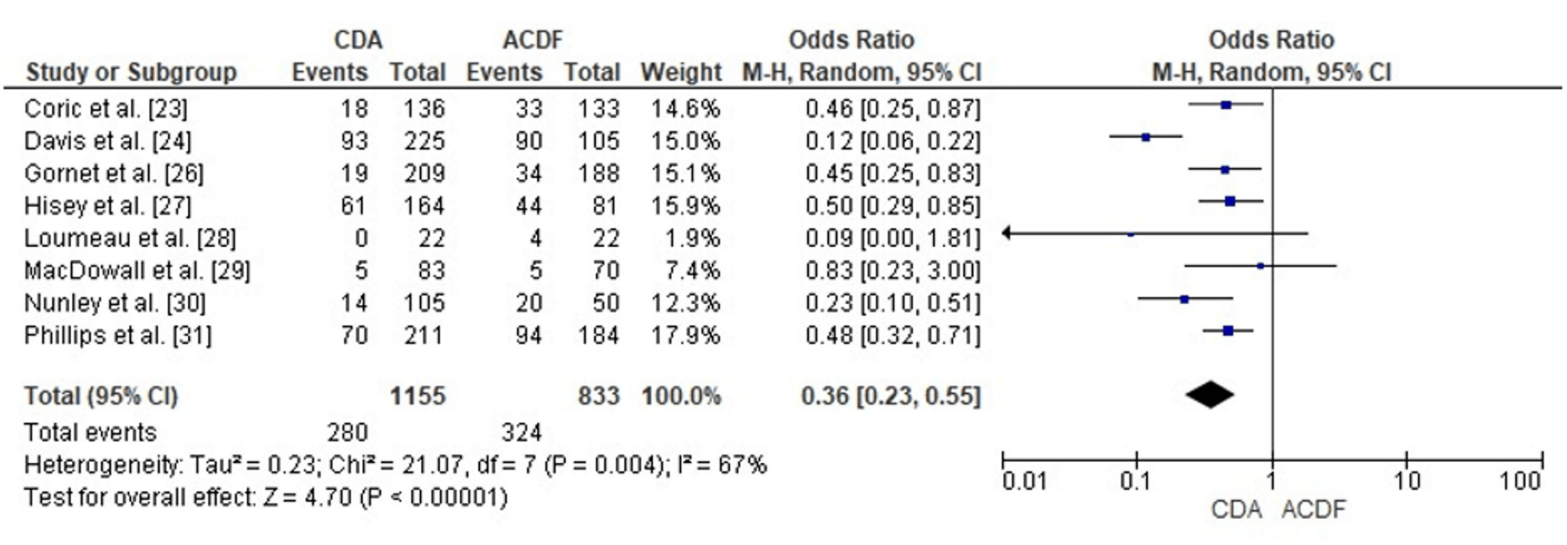
Forest plot comparing CDA and ACDF for ASD CDA: Cervical Disc Arthroplasty; ACDF: Anterior Cervical Discectomy and Fusion; ASD: Adjacent Segment Disease; OR: Odds Ratio; CI: Confidence Interval Source: [23,24,26–31]

The forest plot analysis evaluated ASD occurrence following CDA compared with ACDF. Pooled OR measured 0.36 (95% CI: 0.23 to 0.55), demonstrating statistically significant ASD risk reduction through CDA (p < 0.00001). Moderate heterogeneity characterized the studies (I² = 67%), possibly reflecting variations in study duration, prosthesis design, or patient selection parameters. The findings indicate substantially decreased ASD risk associated with CDA relative to ACDF (Figure 11).

Publication Bias Assessment for ASD

**Figure 12:**
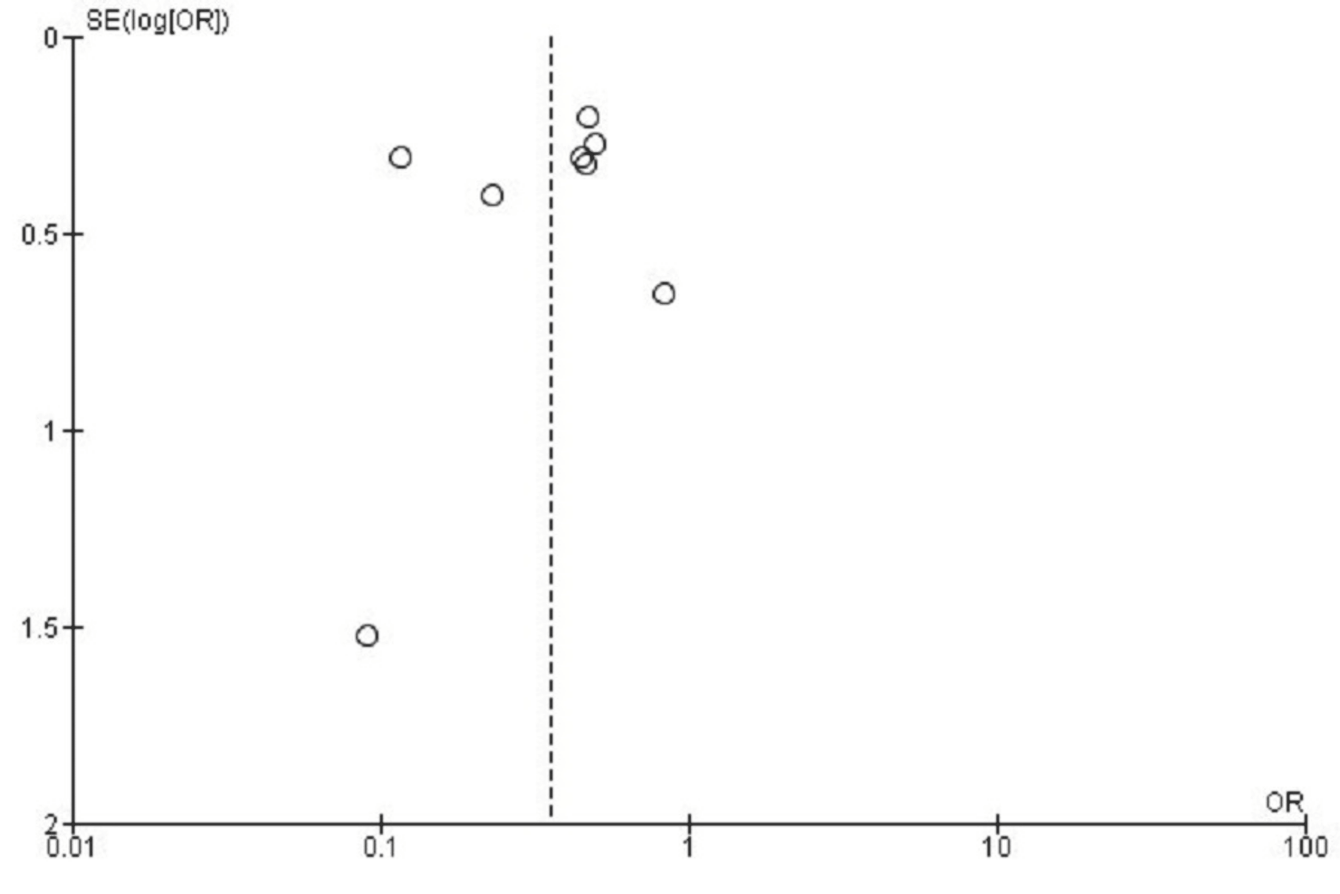
Funnel plot assessing publication bias for ASD outcomes in studies comparing CDA and ACDF CDA: Cervical Disc Arthroplasty; ACDF: Anterior Cervical Discectomy and Fusion; ASD: Adjacent Segment Disease; OR: Odds Ratio; SE: Standard Error

The assessment of the funnel plot for ASD outcomes demonstrated minor asymmetrical distribution, suggesting possible heterogeneity or the influence of small-study effects. The statistical evaluation conducted using Egger's test showed no evidence of significant publication bias (p > 0.05), supporting the robustness of the reported findings (Figure 12).

Discussion

The ongoing evaluation of CDA versus ACDF represents a fundamental consideration in contemporary spinal surgical practice. Despite CDA's emergence as a motion-preserving treatment option, ACDF continues to demonstrate established efficacy as a standard intervention. This meta-analysis provides a comprehensive evaluation of these surgical approaches and examines the consistency of their outcomes with existing literature.

The pooled analysis revealed that CDA demonstrated statistical superiority in improving neck disability based on NDI measurements (SMD = -0.54, 95% CI: -1.00 to -0.08, p = 0.02), although considerable heterogeneity was present (I² = 89%). This observation corresponds with Rožanković et al.'s findings of enhanced NDI outcomes in CDA patients over extended periods [33], while Radcliff et al. similarly documented sustained favorable NDI results through seven-year follow-up [32]. Conversely, MacDowall et al. [29] found comparable long-term NDI outcomes between treatment groups, indicating that CDA's advantages might be restricted to specific patient cohorts.

Analysis of neck pain outcomes (VAS) demonstrated significant benefits associated with CDA (SMD = -0.58, 95% CI: -0.98 to -0.18, p = 0.004), despite substantial heterogeneity (I² = 86%). These findings correspond with observations by Radcliff et al. and Davis et al., both documenting superior neck pain amelioration among CDA recipients [24,32]. Nevertheless, MacDowall et al. documented equivalent VAS neck measurements between treatment arms during extended follow-up, indicating that CDA's effectiveness for neck pain might be contingent upon specific implant designs or selected patient characteristics [29].

Evaluation of arm pain (VAS) revealed statistically meaningful benefits favoring CDA (SMD = -0.44, 95% CI: -0.75 to -0.13, p = 0.006), accompanied by moderate heterogeneity (I² = 76%). Both Gornet et al. and Davis et al. documented superior arm pain amelioration among CDA patients [24,26]. Yet, MacDowall et al. [29] demonstrated equivalent arm pain results, supporting the conclusion that each surgical approach achieves adequate neural decompression.

Reoperation frequency emerged as a critical distinction, with CDA demonstrating substantially reduced risk (OR = 0.40, 95% CI: 0.24 to 0.66, p = 0.0004). These results corroborate extended follow-up data from Gornet et al. and Coric et al., who documented decreased subsequent surgical interventions among CDA recipients [23,26]. However, MacDowall et al. [29] found no meaningful variation in reoperation frequency, suggesting that the advantages might correlate with implant characteristics or the length of the observation period.

ASD occurrence demonstrated significantly reduced frequency among CDA patients (OR = 0.36, 95% CI: 0.23 to 0.55, p < 0.00001). These results correspond with Phillips et al. and Hisey et al.'s documentation of decreased radiographic and symptomatic ASD among CDA recipients [31,27]. Yet, MacDowall et al. found no meaningful variation in ASD frequency, prompting consideration regarding extended disease progression and appropriate patient selection [29].

Funnel plot variations across measured outcomes indicate possible heterogeneity attributable to methodological differences, patient selection protocols, and implant variations. The evidence indicates that CDA provides benefits relative to ACDF, especially regarding functional neck outcomes, pain amelioration, and subsequent surgery prevention. Nevertheless, inter-trial variations emphasize the necessity for consistent implant specifications and patient selection parameters in subsequent investigations.

Limitations

The heterogeneity across included studies regarding implant designs, observation periods, and outcome parameters constitutes a primary limitation of this meta-analysis. Pooled estimates may be affected by non-uniform documentation of extended follow-up data and differences in operative approaches. Furthermore, the absence of blinding protocols and industry funding involvement in multiple trials presents possible sources of bias.

## Conclusions

The present meta-analysis reveals that CDA provides enhanced outcomes relative to anterior ACDF regarding neck and arm pain alleviation, functional improvement, diminished reoperation frequencies, and decreased ASD occurrence. Although both interventions demonstrate effectiveness, CDA represents an optimal choice for carefully selected individuals, especially those prioritizing motion preservation and extended durability. Despite apparent long-term CDA advantages, existing evidence faces limitations from heterogeneity in prosthesis specifications, follow-up periods, and outcome criteria. Additional high-quality, extended randomized investigations employing standardized surgical techniques and outcome measurements remain essential to establish CDA's definitive clinical role.
